# Using the changes of several simple anthropometric indices to predict the occurrence of metabolic syndrome: Findings from medically under-resourced communities in rural China

**DOI:** 10.3389/fendo.2022.1014541

**Published:** 2022-10-17

**Authors:** Qiyu Li, Pengbo Wang, Guangxiao Li, Ye Chang, Xiaofan Guo, Yingxian Sun, Xingang Zhang

**Affiliations:** ^1^ Department of Cardiology, The First Hospital of China Medical University, Shenyang, China; ^2^ Department of Medical Record Management, The First Hospital of China Medical University, Shenyang, China

**Keywords:** anthropometric indices, metabolic syndrome, epidemiology, central obesity, public health

## Abstract

**Background:**

Various anthropometric indices have been proved to be useful to predict metabolic syndrome(MetS), but the association between changes in anthropometric indices and the onset of MetS is unclear. This study selected six indices that are easy to measure and calculate in daily life and evaluated the relationships.

**Methods:**

We established a prospective cohort in rural China during 2012-2013 and involved 5,221 participants without MetS. The follow-up visit was conducted in 2015 to repeat anthropometric indices measurements and assess MetS onset. Binary logistic regression model was used to calculate the association between changes in anthropometric indices and MetS onset. Receiver operating characteristic (ROC) curve was drawn to compare their abilities in MetS prediction.

**Results:**

Over a median follow-up time of 2.42 years, 1,367 participants (26.2%) developed MetS. The increase in all the six indices is associated with an increased risk of MetS. Changes in WC and WHtR are the strongest predictors, with a 5 cm increase in WC and a 0.025 increase in WHtR giving the best prediction of MetS onset.

**Conclusions:**

People should be aware of changes in these six anthropometric indices in daily life, as their increase is closely related to an increased risk of MetS, especially WC and WHtR. We recommend an increase of 5 cm in WC and 0.025 in WHtR as the optimal cut-off for the MetS prediction.

## Introduction

Metabolic syndrome (MetS) is a cluster of the most dangerous heart attack risk factors including central (abdominal) obesity, raised triglycerides, elevated blood pressure, reduced high density lipoprotein cholesterol and raised fasting plasma glucose (1). According to data from 2018, an estimated one-quarter of the global population suffers MetS (2). MetS is strongly associated not only with cardiovascular disease, cerebrovascular disease and kidney disease (3–5), but also with an increased risk of cardiovascular and all-cause mortality (6, 7). Thus, early detection and timely intervention of MetS is essential in preventing the occurrence and development of various chronic diseases and deaths.

There are numerous indices that can reflect the degree of obesity, including directly measured indicators such as body weight(BW), waist circumference(WC), hip circumference(HC) (8) and calculated ones such as body mass index(BMI), waist-hip ratio(WHR), waist-height ratio(WHtR), visceral adiposity index(VAI), body adiposity index(BRI), a body shape index (ABSI), etc (9, 10). It has been found that various obesity-related indices are highly correlated with MetS and can be used to predict its occurrence (11, 12). However, these indices can only reflect the obesity status at a given moment, not the dynamic changes. Currently, there are no studies describing the relationship between changes in anthropometric indices and MetS onset.

This study selected several anthropometric indices (including BW, WC, HC, BMI, WHR, WHtR) which were easy to measure and calculate in a clinical setting, aiming to find out the association between the changes in them and MetS onset.

## Method

### Study population

The study population was 11,956 residents aged ≥35 involved in the Northeast Rural Cardiovascular Health Study (NCRCHS). The cohort was established in Liaoning Province from January 2012 to August 2013 and has been described in detail in our previous protocols (13, 14). Briefly, by multi-stage random cluster sampling, 26 villages from 3 counties of Liaoning Province were selected randomly, all residents aged ≥35 in the villages were invited to join and the ones who finished the baseline investigation were selected. Baseline information on the subjects was collected through questionnaires, physical measurements, and blood biochemical tests. The follow-up visit was conducted in 2015 to repeat physical measurements and blood biochemical tests.

At baseline, 212 subjects with incomplete data and 4,914 subjects diagnosed with MetS were excluded. At the time of follow-up in 2015, 1,166 subjects had missed follow-up, 93 subjects had died, and 350 subjects who failed to complete blood chemistry tests or physical measurements were also excluded. Ultimately, a total of 5,221 subjects were involved in the study. This study was approved by the Ethics Committee of China Medical University (Shenyang, China) and all participants gave written informed consent.

### Study variables and definitions

Subjects’ personal information of age, gender, current smoking and drinking status, regular exercise, vegetables consumption frequency (whether ≥3kg/week or not) and greasy food consumption frequency (whether ≥4times/week or not) was recorded through face-to-face questionnaires. Participants were asked to rest for at least five minutes in a quiet room with a moderate temperature before blood pressure measurements, then place their bare upper arms at the level of the heart. Trained personnel measured blood pressure using standard electronic sphygmomanometers (HEM-907; Omron, Tokyo, Japan). Blood pressure was measured 3 times at 2 minute intervals and the average of the measurements was recorded for subsequent analysis. Participants were then weighed wearing only light clothing and no shoes, and the results were accurate to 0.1 kg and 0.1 cm. Waist circumference (WC) was measured at the umbilicus and horizontal hip circumference (HC) was measured at the most prominent part of the buttocks using a tape measure to nearest 0.1cm. All participants were required to fast for at least 12 hours prior to the blood biochemistry test, and their venous blood was collected at the elbow the following morning. Blood samples were added to a vacuum tube containing anticoagulant and plasma was obtained by centrifugation. Triglyceride (TG), high density lipoprotein cholesterol (HDL-C)), fasting blood glucose (FPG) were automatically analyzed by machines (Olympus AU 640, Tokyo, Japan).

Metabolic syndrome (Mets) was defined as a combination of 3 or more of the following 5 criteria: (a) central (abdominal) obesity: defined as WC ≥85 cm for males and ≥80 cm for females; (b) BP ≥130/85 mmHg or current use of antihypertensive drugs; (c) FPG ≥5.6 mmol/L or current use of antihyperglycemic agents; (d) TG ≥1.7 mmol/L; (e) HDL-C <1.0 mmol/L for males and <1.3 mmol/L for females, according to the harmonized International Diabetes Federation criteria. BMI was calculated as weight (kg) divided by height (meters) squared. WHR and WHtR were calculated by dividing WC(cm) by HC(cm) or height (cm).

The change of BW was calculated as BW measured in follow-up visit minus BW measured at baseline, recorded as delta BW(△BW). The changes of WC, HC, BMI, WHR, WHtR were calculated in the same way, recorded as △WC, △HC, △BMI, △WHR and △WHtR, respectively. They were then standardized and recorded as z-△BW, z-△WC, z-△HC, z-△BMI, z-△WHR and z-△WHtR and divided into T1, T2 and T3 three groups based on tertiles.

### Statistical analysis

Descriptive statistics was performed for variables, continuous variables were expressed as mean ± standard deviation, categorical variables were expressed as numbers (percentages). Differences were assessed using nonparametric and chi-squared tests. The binary logistic regression model was established to calculate risk ratios (RRs) and 95% confidence intervals (CIs) of the risk of MetS. Adjusted variables were diagnosed by collinearity, variance inflation factor (VIF) <10 was acceptable. Receiver operating characteristic (ROC) curve analysis was used to compare the diagnostic performance of changes of anthropometric indices for MetS. The data analysis above were performed using IBM SPSS statistical software, version 26 (IBM Corporation, Armonk, New York, NY, USA) and R statistical software package (http://www.r-project.org, R Foundation), two-tailed P-value below 0.05 was regarded statistically significant.

## Results

### Baseline characteristics of the study population

Of the 5,221 subjects involved, the mean age was 52.6 years and 49.8 percent were male. Over a median follow-up of 2.42 years, 1,367 participants developed MetS, a rate of 26.2 percent. Subjects with MetS were more likely to have more risk factors such as being older, having higher SBP, DBP, TG, FPG, lower HDL-C, and not taking regular exercise, as detailed in **Table 1**.

**Table 1:** Baseline characteristics of subjects grouped according to whether developed MetS at follow-up.

	Total (n = 5221)	MetS (n = 1367)	non-MetS (n = 3854)	P value
Male(%)	2600 (49.8)	672 (49.2)	1928 (50.0)	0.604
Age(Year)	52.6 ± 10.2	54.0 ± 10.0	52.2 ± 10.3	<0.001
Ethnicity of Han(%)	4923 (94.3)	1290 (94.4)	3633 (94.2)	0.232
Current smoking(%)	1975 (37.8)	527 (38.6)	1448 (37.5)	0.504
Current drinking(%)	3966 (76.0)	1017 (74.4)	2949 (76.4)	0.129
Regular exercise(%)	4240 (81.2)	1081(79.1)	3159 (81.9)	0.023
Vegetables consumption frequency(%)	3415 (65.4)	898 (65.7)	2517 (65.2)	0.781
Greasy food consumption frequency(%)	1043 (20.0)	269 (19.7)	774 (20.1)	0.76
TG(mmol/L)	1.1 ± 0.8	1.3 ± 1.1	1.1 ± 0.6	<0.001
HDL-C(mmol/L)	1.5 ± 0.4	1.5 ± 0.4	1.5 ± 0.4	<0.001
SBP(mmHg)	135 ± 22	141 ± 23	133 ± 21	<0.001
DBP(mmHg)	79 ± 11	82 ± 11	78 ± 11	<0.001
FPG (mmol/L)	5.5 ± 1.0	5.6 ± 1.2	5.4 ± 1.0	<0.001
BW (kg)	60.8 ± 9.9	64.2 ± 10.3	59.6 ± 9.4	<0.001
Height (cm)	160.8 ± 8.0	161.0 ± 8.3	160.8 ± 7.9	0.463
WC(cm)	78.1 ± 8.4	81.6 ± 8.4	76.9 ± 8.1	<0.001
HC(cm)	93.8 ± 6.7	95.5 ± 7.3	93.2 ± 6.4	<0.001
WHR	0.83 ± 0.07	0.83 ± 0.07	0.86 ± 0.09	<0.001
WHtR	0.49 ± 0.05	0.51 ± 0.05	0.48 ± 0.05	<0.001
BMI(kg/m^2^)	23.5 ± 3.2	24.7 ± 3.1	23.0 ± 3.1	<0.001

TG, total triglyceride; HDL-C, high density lipoprotein cholesterol; SBP, systolic blood pressure; DBP, diastolic blood pressure; FPG, fasting plasma glucose; BW, body weight; WC, waist circumference; HC, hip circumference; WHR, waist-hip-ratio; WHtR, waist-height-ratio; BMI, body mass index.

### Association between the changes in the six anthropometric indices and MetS risk

**Table 2** shows the results of binary logistic regression with the listed crude RRs, adjusted RRs, 95% CIs and p-values. The fully adjusted model includes confounding variables of age, sex, nation, current smoking, current drinking, regular exercise, frequent vegetables consumption, frequent greasy food consumption and BMI grades at baseline (<18.5kg/m^2^, 18.5-25kg/m^2^, 25-30kg/m^2^, ≥30kg/m^2^). According to the results, increases in all six anthropometric indices were associated with an increased risk of MetS. Each 1-SD increment in BW, WC, HC, BMI, WHR and WHtR was associated with a 87%, 102%, 58%, 74%, 41% and 100% increased risk of incident MetS, respectively. When they are treated as categorical variables, with T2 (subjects with steady indices) as a reference, the risk of MetS was significantly increased in T3 (subjects with increased indices) and decreased in the T1 (subjects with decreased indices group), detailed data seen in **Table 2**.

**Table 2:** Crude and multivariate-adjusted risk ratios and 95% confidence intervals of binary logistics regression model.

	Variables	crude RR (95%CI)	p value	adjusted RR (95%CI)	p value
z-△BW	per SD increment	1.550 (1.443-1.664)	<0.001	1.868 (1.725-2.022)	<0.001
	T1	0.720 (0.610-0.850)	<0.001	0.562 (0.471-0.671)	<0.001
	T2	Ref	–	Ref	–
	T3	1.960 (1.691-2.272)	<0.001	2.225 (1.903-2.602)	<0.001
z-△WC	per SD increment	1.868 (1.738-2.008)	<0.001	2.019 (1.871-2.179)	<0.001
	T1	0.546 (0.459-0.650)	<0.001	0.509 (0.425-0.611)	<0.001
	T2	Ref	–	Ref	–
	T3	2.269 (1.960-2.627)	<0.001	2.480 (2.126-2.893)	<0.001
z-△HC	per SD increment	1.402 (1.312-1.498)	<0.001	1.583 (1.471-1.702)	<0.001
	T1	0.816 (0.695-0.957)	0.013	0.720 (0.609-0.851)	<0.001
	T2	Ref	–	Ref	–
	T3	1.565 (1.350-1.815)	<0.001	1.766 (1.511-2.065)	<0.001
z-△BMI	per SD increment	1.376 (1.289-1.468)	<0.001	1.735 (1.611-1.867)	<0.001
	T1	0.971 (0.828-1.139)	0.721	0.736 (0.621-0.873)	<0.001
	T2	Ref	–	Ref	–
	T3	1.714 (1.476-1.992)	<0.001	2.007 (1.708-2.359)	<0.001
z-△WHR	per SD increment	1.409 (1.310-1.514)	<0.001	1.405 (1.304-1.515)	<0.001
	T1	0.722 (0.614-0.848)	<0.001	0.734 (0.621-0.867)	0.001
	T2	Ref	–	Ref	–
	T3	1.621 (1.399-1.877)	<0.001	1.637 (1.405-1.908)	0.001
z-△WHtR	per SD increment	1.867 (1.737-2.006)	<0.001	2.007 (1.860-2.165)	<0.001
	T1	0.544 (0.457-0.647)	<0.001	0.506 (0.422-0.607)	<0.001
	T2	Ref	–	Ref	–
	T3	2.218 (1.916-2.567)	<0.001	2.393 (2.052-2.790)	<0.001

Adjusted confounding variables include: age, sex, nation, current smoking, current drinking, regular exercise, vegetables consumption frequency, greasy food consumption frequency and grades of BMI.

### Variations in the six indices can improve the discriminative ability of the conventional models

**Figure 1:**
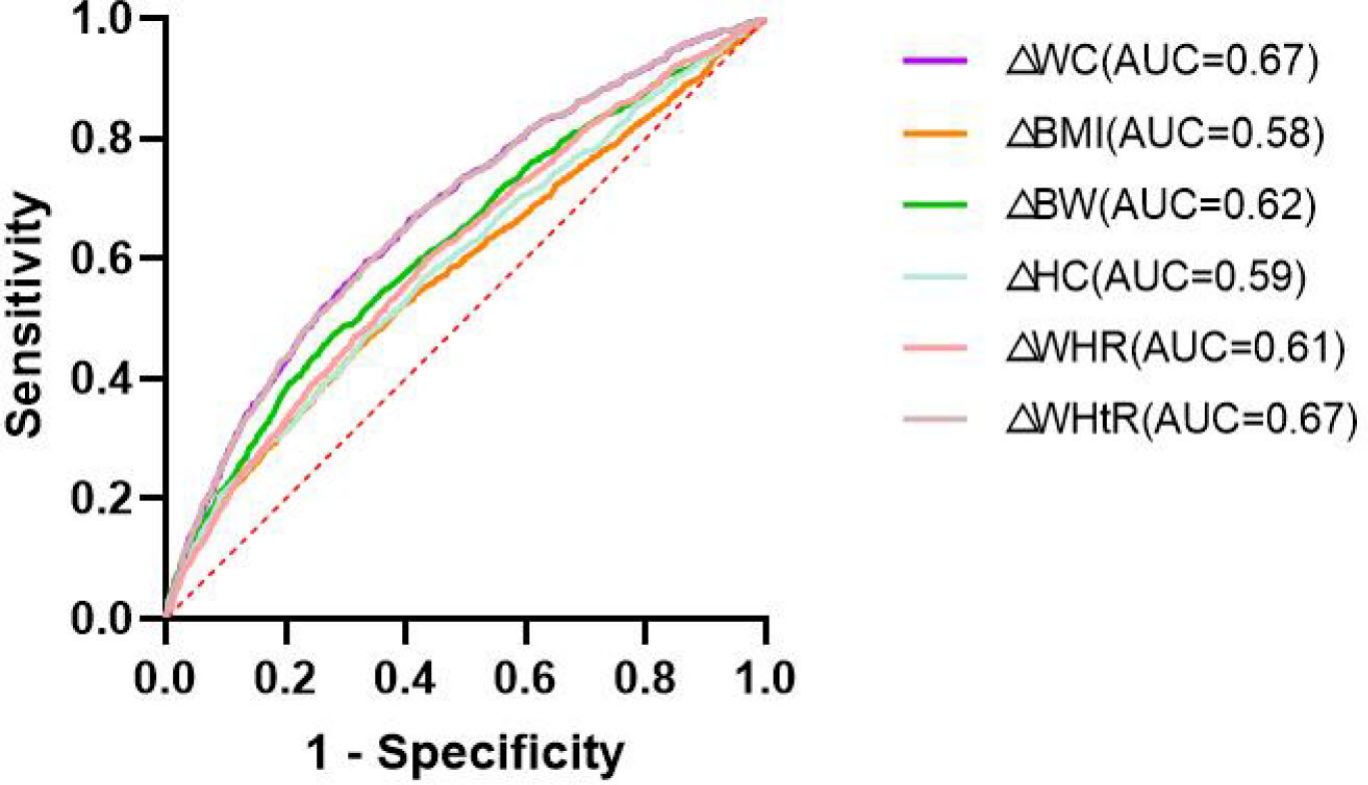
The areas under the curves (AUC).

**Figure 2:**
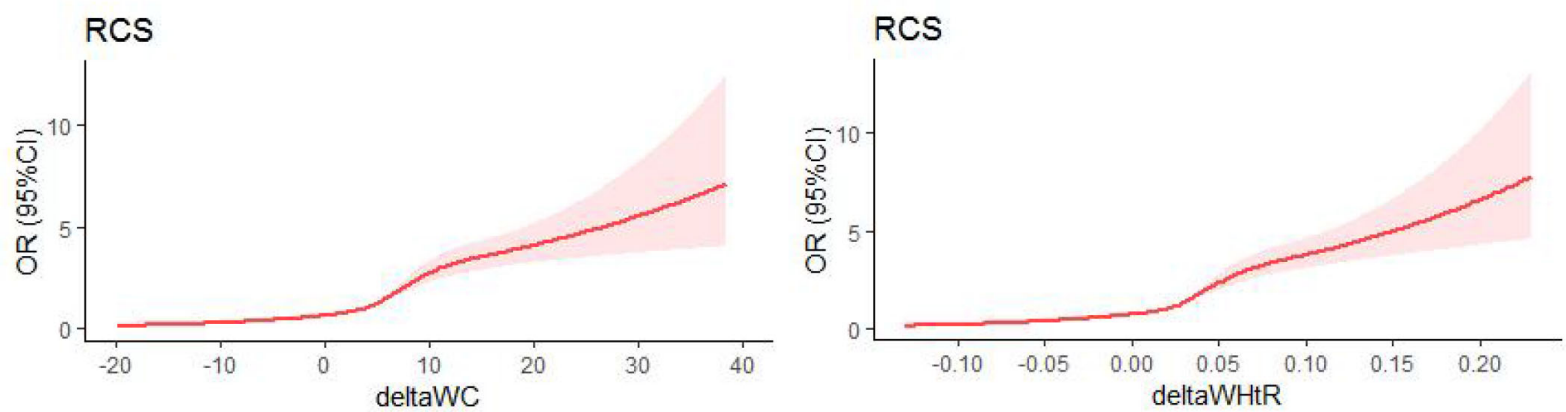
Restricted cubic splines(RCS) (knot=5).

The areas under receiver operating characteristic curves (AUC) and the values of optimal Cut-Offs, Youden Index, sensitivity, specificity were listed in **Table 3**. The AUC of WC and WHtR were the largest, at 0.672 (p < 0.001), seen in **Figure 1**. The optimal cut-off values are 5.025 and 0.025, respectively. The restricted cubic splines(RCS) (knot=5) for deltaWC and deltaWHtR were showed in **Figure 2**, the inflection points of the sharp rise in OR values are around 5 and 0.025, respectively. Restrictive cubic splines for the remaining four indices can be found in **Supplementary Figure S1** if interested.

**Table 3:** Receiver operating characteristic (ROC) curve related parameters.

Variables	AUC	Optimal Cut-Offs	Youden Index	Sensitivity(%)	Specificity(%)	p value
△BW	0.622	1.463	0.193	47	72.3	<0.001
△WC	0.672	5.025	0.26	59.4	66.6	<0.001
△HC	0.590	2.063	0.138	47.5	66.4	<0.001
△BMI	0.577	0.663	0.131	51.3	61.8	<0.001
△WHR	0.607	0.303	0.165	59.9	56.6	<0.001
△WHtR	0.672	0.025	0.258	67.5	58.3	<0.001

### Subgroup analysis

**Table 4** shows the results of the subgroup analysis, where increases in all six anthropometric indices increased the risk of MetS regardless of age, sex, or abdominal obesity status. And for male or non-abdominal obese ones(WC ≥85 cm for males and ≥80 cm for females), the risk would increase more. E.g. per 1-SD increase in WC would have a 128% risk increase (RR: 2.284; 95%CI: 2.038-2.560) in males, while only a 79% risk increasing (RR: 1.788; 95%CI: 1.611-1.985) in female. Similar differences between males and females, obese and non-abdominal obese, were also observed in the remaining five indices.

**Table 4:** Subgroup analyses by age, sex and central obesity (abdominal) status at baseline.

	variables	aRR (95%CI)	p-value
Age (baseline)			
35≤age<60	z-△BW	1.904 (1.734-2.090)	<0.001
	z-△WC	1.969 (1.800-2.153)	<0.001
	z-△HC	1.561 (1.430-1.705)	<0.001
	z-△BMI	1.734 (1.588-1.892)	<0.001
	z-△WHR	1.434 (1.308-1.572)	<0.001
	z-△WHtR	1.959 (1.791-2.143)	<0.001
age≥60	z-△BW	1.771 (1.527-2.054)	<0.001
	z-△WC	2.109 (1.821-2.443)	<0.001
	z-△HC	1.601 (1.404-1.826)	<0.001
	z-△BMI	1.728 (1.504-1.984)	<0.001
	z-△WHR	1.337 (1.174-1.523)	<0.001
	z-△WHtR	2.087 (1.804-2.415)	<0.001
Sex			
Male	z-△BW	2.000 (1.780-2.247)	<0.001
	z-△WC	2.284 (2.038-2.560)	<0.001
	z-△HC	1.721 (1.555-1.903)	<0.001
	z-△BMI	1.926 (1.728-2.146)	<0.001
	z-△WHR	1.408 (1.262-1.570)	<0.001
	z-△WHtR	2.343 (2.083-2.636)	<0.001
Female	z-△BW	1.744 (1.563-1.946)	<0.001
	z-△WC	1.788 (1.611-1.985)	<0.001
	z-△HC	1.432 (1.286-1.594)	<0.001
	z-△BMI	1.569 (1.416-1.738)	<0.001
	z-△WHR	1.379 (1.243-1.531)	<0.001
	z-△WHtR	1.747 (1.580-1.933)	<0.001
Abdominal obesity (baseline)			
yes	z-△BW	1.680 (1.436-1.965)	<0.001
	z-△WC	1.899 (1.644-2.193)	<0.001
	z-△HC	1.394 (1.217-1.597)	<0.001
	z-△BMI	1.550 (1.356-1.772)	<0.001
	z-△WHR	1.537 (1.325-1.782)	<0.001
	z-△WHtR	1.900 (1.644-2.196)	<0.001
no	z-△BW	2.007(1.825-2.208)	<0.001
	z-△WC	2.438 (2.221-2.688)	<0.001
	z-△HC	1.714 (1.568-1.873)	<0.001
	z-△BMI	1.852 (1.693-2.027)	<0.001
	z-△WHR	1.517 (1.381-1.667)	<0.001
	z-△WHtR	2.418 (2.194-2.666)	<0.001

## Discussion

This study, a prospective cohort study in rural northeastern China, demonstrated a positive association between increased BW, WC, HC, BMI, WHR and WHtR and MetS occurrence. Compared to subjects with stable anthropometric indices, subjects with decreased and increased indices were significantly associated with decreased and increased MetS risk, respectively. This conclusion holds for all of the six measures mentioned above, and the relationship is even more pronounced for subjects who are either male or non-abdominal obese. Previous studies have indicated an association between MetS and these anthropometric indices (15–17). However, these are cross-sectional analyses and can only tell the relation to current obesity status. At present, there are few studies focusing on the variability of anthropometric indices. A South Korean study included 932 North Korean refugees and drew a conclusion that the risk of MetS in the weight gain group (≥5kg) was nearly 2 times that of that in the non-weight gain group (<5kg) (18), which is similar to our findings. However, our study was conducted in a general population with a larger sample size and treated changes in weight as a continuous variable, further illustrating the linear relationship between increased weight and increased risk of METS. The study by Liu et al. involved 931 adolescent subjects and concluded that increasing BMI increased the risk of METS in adulthood through 5 years of follow-up (19). Another study, involving 789 women over the age of 40 at the time of delivery, showed that weight changes during pregnancy were associated with MetS incidence (20). The cohort study in Guizhou, China, which demonstrated an effect of changes in BMI, WHR and WC on dyslipidemia, may also support our conclusions from the side (21).

In the diagnostic sensitivity analysis, △WC and △WHtR showed the best diagnostic value for MetS, which was similar to the conclusions from previous cross-sectional studies (11, 22, 23). These studies demonstrate the superiority of baseline WC in predicting MetS, and our study further demonstrates that changes in WC also have an advantage over other indices. Subsequently, by plotting the ROC curve and computing the area under the curve while plotting the constrained cubic spline, we found that a 5 cm increase in WC or 0.025 increase in WHtR is the best cut-off for predicting the occurrence of MetS.

The pathophysiological basis of MetS can be explained by insulin resistance, excess fatty acid flow, and a proinflammatory state (24) that causes damage to the vascular endothelial system (25), leading to cardiovascular and cerebrovascular disease (26, 27). Processes that occur *in vivo*, such as atherosclerosis of vessel walls, are difficult to detect in time. Timely detection of metabolic disorders requires regular monitoring of blood pressure, glucose and lipids. But this is difficult to achieve, especially in places where medical resources are relatively scarce and residents are less health-conscious. In this context, it is of particular interest to predict the occurrence of MetS by focusing on simple anthropometric indices changes. It has been suggested that weight loss is an effective measure for MetS prevention (28), and the results of this study further support this view.

There are still some gaps in this study. First, we do not include some novel anthropometric indices, such as BRI, VAI, ABSI, etc. Some studies have argued that they provide a better description of central obesity than traditional indices (15, 29). But from our point of view, perhaps these novel indices have certain advantages, but their calculations are too complicated to gain wide attention and applications in daily life. Second, we measured WC at horizontal plane of umbilicus, which is not compliant with IDF recommendations (at horizontal plane of midway between the inferior margin of the ribs and the superior border of the iliac crest). Our study was conducted in rural areas of northeastern China, where we know that local residents typically have low levels of education and poor health awareness, and where IDF-recommended WC measurements may be difficult to generalize and standardize. Our study aims to generalize the use of observing changes in simple anthropometric indices to alert subjects to the occurrence of MetS in their daily lives. With this in mind, measuring the WC in the horizontal plane of the umbilicus seems to be an easier and better option to implement. More importantly, it has been demonstrated that measuring the WC in the horizontal plane of the umbilicus and in the horizontal plane of the midpoint makes little difference in the measurement results (16) and the diagnosis of MetS (17). Third, only one follow-up was conducted in this study, and changes in anthropometric indices and MetS occurrence rates were collected simultaneously, so the causal relationship is not clear. In addition, our study was limited to rural populations in northeastern China and did not fully account for differences among ethnic groups. Whether the conclusions of our study can be broadly applied to different ethnic groups still requires further study.

## Conclusion

The increments in all the six anthropometric indices mentioned in this paper are significantly associated with MetS onset. Of these, WC and WHtR have the highest predicted values. Therefore, we recommend that everyone, especially those in medically under-resourced communities, pay timely attention to changes in these indices as they are highly reliable markers of MetS onset. Based on the results of this study, we recommend an increase of 5 cm in WC and 0.025 in WHtR as a predicted marker for the occurrence of MetS.

## Data availability statement

The raw data supporting the conclusions of this article will be made available by the authors, without undue reservation.

## Author contributions

YS and XZ directed the design of study. XG was responsible for the study conduct. GL and PW was responsible for methodology. QL analyzed the data and wrote the manuscript. YC and GL corrected the manuscript. All authors contributed to the article and approved the submitted version.

## Funding

This research was supported by following funding: The National Key Research and Development Program from the Ministry of Science and Technology of China (Project Grant # 2018YFC1312400, Sub-project Grant # 2018YFC1312403); The Science and Technology Program of Liaoning Province, China (Grant #2020JH1/10300002); The National Key Research and Development Program from the Ministry of Science and Technology of China (Project Grant # 2016YFC1301305); The National Key Research and Development Program from the Ministry of Science and Technology of China (Project Grant #2012BAJ18B00, Sub-project Grant # 2012BAJ18B08);The thirteenth Five-Year National Key R&D Plan (Project Grant #2016YFC1301305).

## Conflict of interest

The authors declare that the research was conducted in the absence of any commercial or financial relationships that could be construed as a potential conflict of interest.

## Publisher’s note

All claims expressed in this article are solely those of the authors and do not necessarily represent those of their affiliated organizations, or those of the publisher, the editors and the reviewers. Any product that may be evaluated in this article, or claim that may be made by its manufacturer, is not guaranteed or endorsed by the publisher.

## Abbreviations

metabolic syndrome, MetS; body weight, BW; waist circumference, WC; hip circumference, HC; body mass index, BMI; waist-hip ratio, WHR, waist-height ratio, WHtR, visceral adiposity index, VAI; body adiposity index, BRI; a body shape index, ABSI; Receiver operating characteristic, ROC; areas under the curve, AUC; triglyceride, TG; high density lipoprotein cholesterol, HDL-C; fasting blood glucose, FPG; risk ratio, RR; aRR, adjusted risk ratio; confidence interval (CI); variance inflation factor, VIF.
