# Supplementary material for: Using the changes of several simple anthropometric indices to predict the occurrence of metabolic syndrome: Findings from medically under-resourced communities in rural China
Source: Front Endocrinol (Lausanne). 2022 Oct 17;13:1014541. doi: 10.3389/fendo.2022.1014541 (PMC9618802; doi:10.3389/fendo.2022.1014541)

Supplementary Figure 1 Restricted cubic splines(RCS) for deltaBW, deltaHC, deltaBMI and deltaWHR

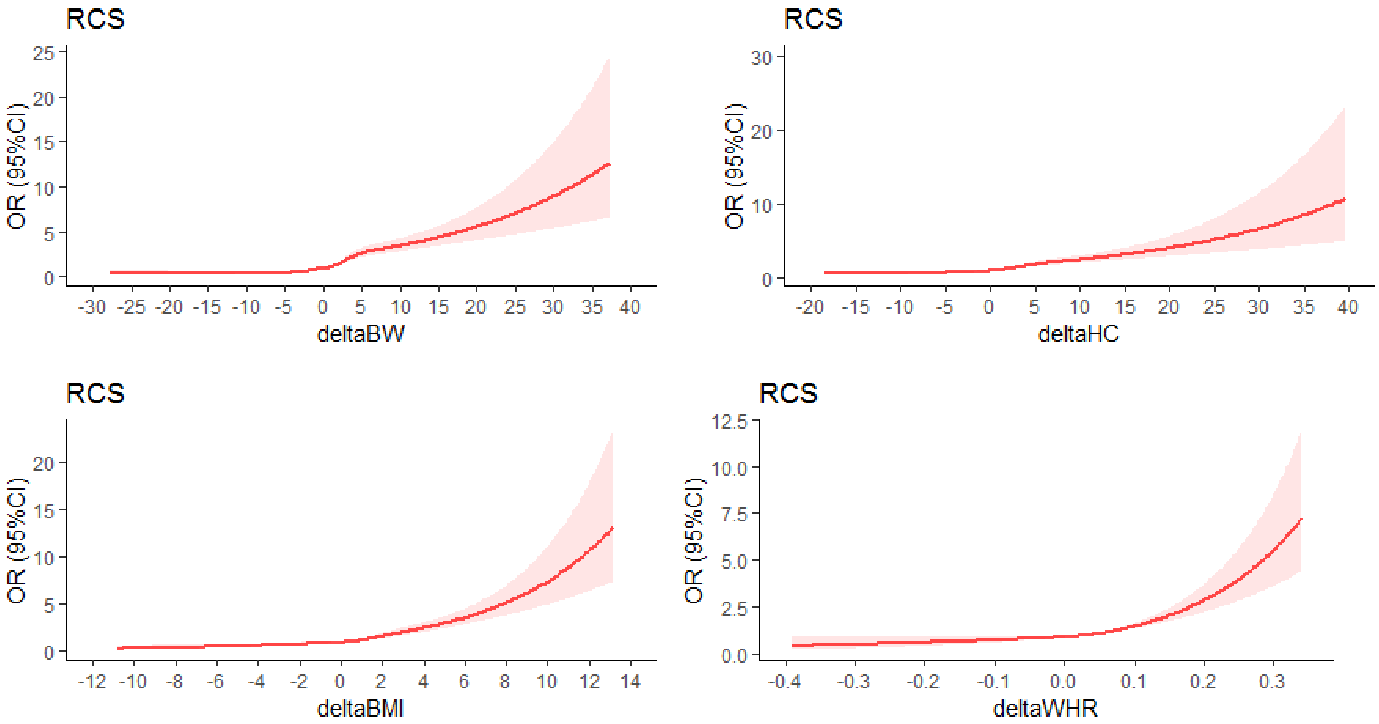

Supplement: Supplementary file 1 [file Image_1.pdf]
